# Cardiac Arrhythmia: Molecular Mechanisms and Therapeutic Strategies

**DOI:** 10.3390/ijms252413253

**Published:** 2024-12-10

**Authors:** Yosuke Okamoto, Kunichika Tsumoto

**Affiliations:** 1 Department of Cell Physiology, Akita University Graduate School of Medicine, 1-1-1, Hondo, Akita 010-8543, Japan; 2 Department of Physiology II, Kanazawa Medical University, 1-1 Daigaku, Uchinada, Ishikawa 920-0293, Japan

Arrhythmias are divided into supraventricular and ventricular, depending on where they originate. Supraventricular arrhythmias are characterized by the presence of extra-cardiac complications, while ventricular arrhythmias have a high mortality rate.

In this Special Issue, all of the papers on supraventricular arrhythmias, with the exception of Dr. Kayser’s paper, are on the theme of atrial fibrillation (AF). AF is the most frequent of all arrhythmias and can be described as the king of arrhythmias.

In a review article, Iwamiya et al. [1] explain the macroscopic anatomy, histological findings, and molecular mechanisms of AF, using “heterogeneity” as a keyword, and also explain the treatment strategies. Most cases of idiopathic AF are caused by electrical re-entry between the pulmonary veins (PVs) and left atrium and are diagnosed as sustained arrhythmia [2]. The heterogeneity of thickness of the heart wall and the muscle fiber arrangement between the PVs and atrium are some of the factors that cause and sustain AF. In particular, the muscle bundles between same-side PVs are called ‘carina’ and are an important target for radiofrequency ablation [3]. When AF becomes persistent or chronic, atrial fibrosis becomes a major factor in the pathological condition [4]. Cultured cell study suggests that intercellular communication between myofibroblasts and cardiomyocytes is the cause of electrophysiological heterogeneity inside the atrium [5]. Recent advances in imaging technology have made it possible to visualize fibrosis using MRI non-invasively [6]. Meanwhile, there has also been progress in evaluating fibrosis using electrophysiology with catheter electrodes [7]. Interestingly, the evaluation of fibrosis using MRI and electrophysiology does not overlap. There appears to be a mismatch between structural and functional changes in the extent of fibrosis. Further research is thus necessary for future therapeutic applications in evaluating fibrosis. In particular, there is hope for developing drugs that reduce heterogeneity. Twenty-five years have passed since the discovery of the ‘PV theory’, and the progress of non-drug therapy, percutaneous ablation, is reaching its limits.

Perhaps it would be a good idea to introduce molecular-targeted therapy for AF, which is analogous to cancer treatment. The research by Dr. Igarashi and his colleagues [8] is a comprehensive analysis of gene expression in the pathology of non-paroxysmal AF. This research shows that the PVs lose their characteristics as cardiac tissue and instead transform into tissue similar to the cancer-related microenvironment. It is known that the microenvironment is formed prior to cancer metastasis and invasion [9]. The ectoderm, such as vascular endothelium and mucosal epithelium in the cancer-related microenvironment, is converted into mesenchymal tissue, such as fibroblasts [10]. The heart is an organ that is extremely resistant to cancer, but it is known that severe fibrosis occurs in the atria and PVs of patients with AF [11,12]. In recent years, the relationship between AF and cancer has also been attracting attention [13]. Some reports have already suggested that endothelial-mesenchymal transition occurs in the atria of patients with AF [14,15]. The paper by Igarashi et al. was the first to suggest the possibility that a similar endothelial-mesenchymal transition occurs in the PVs of AF patients.

Dr. Kayser and colleagues [16] studied sinus node dysfunction (SND), namely that of bradycardia. A known single amino acid mutation in G protein-coupled inward rectifying potassium (GIRK) channel has been reported to cause autosomal dominant familial SND [17], and the mutation causes bradycardia. The authors used induced pluripotent stem cell (iPS) technology and gene editing techniques to confirm the phenotype. The iPS cells were differentiated using a specialized method, applying retinoic acid to induce atrial-like cells [18,19]. The cause of the bradycardia was that the GIRK channels were not closing due to a genetic mutation. This discovery is an important report suggesting that selective inhibitors against the channel could be an effective treatment strategy.

For life-threatening arrhythmias such as ventricular tachycardia (VT) and ventricular fibrillation (VF), electrical cardioversion is required for immediate life-saving intervention [20]. In the ventricles where VT (or VF) was induced, it is believed to be in a state in which the excitation wave is circling (or in which the excitation wave is splitting and fusing irregularly) within the tissue [21,22,23]. Electrical cardioversion is performed to reset the ventricles in the VT/VF state. Resetting the cardiac excitable state is a forced switch that accelerates repolarization from a depolarized state to a quiescent state, and the electrical stimulus delivered by the automated external defibrillator (AED) has to propagate the repolarization switch throughout the entire ventricles in an avalanche fashion. The mechanism of successful defibrillation is still unknown, and the study by Himeno et al. [24] may explain part of this defibrillation mechanism. It has been reported that the inactivation of I_NaL_ and of L-type Ca^2+^ current (I_CaL_), the fast component of the delayed rectifier K^+^ channel current (I_Kr_), and the activation of the inward rectifier K^+^ channel current (I_K1_) were the key to repolarization propagation.

Besides, the VT/VF is inducible by the cardiotoxicity (pro-arrhythmogenicity) of drugs [25]. The human ether-à-go-go-related gene (hERG) channel screening is well known in terms of investigating cardiotoxicity in drug development. Dr. Furutani [26] reviews the pharmacological actions of hERG channel inhibitors, the underlying molecular mechanisms, and the clinical implications of these drugs. The hERG channel, which is thought to constitute I_Kr,_ controls the action potential of the human heart by balancing voltage-dependent activation and inactivation. This channel is more active during the repolarization phase than the depolarization phase [27].

For this reason, suppression of this channel causes an increase in the QT interval on the electrocardiogram. Since QT prolongation increases the risk of inducing the VT/VF, unexpected suppression of the hERG channel can cause medical accidents [28]. Unfortunately, the hydrophobic space within the hERG channel is large, and many lipophilic drugs act on it [29].

On the other hand, some Class III antiarrhythmic drugs that inhibit hERG channels have a “facilitating” effect [30] and are used in clinical practice. The fact that an inhibitor is also a “facilitator” is difficult to rationalize, but Furutani’s review article approaches the truth of the molecular mechanism.

As described above, research into arrhythmia is a fascinating field in which basic medicine and clinical application are closely linked. I hope that you will read the articles in this Special Issue that interest you and gain a deeper insight into the arrhythmia research.
